# Assessment of Perceived Barriers to Herpes Zoster Vaccination among Geriatric Primary Care Providers

**DOI:** 10.3390/pharmacy4040030

**Published:** 2016-10-18

**Authors:** Katherine Montag Schafer, Shannon Reidt

**Affiliations:** 1 Department of Family Medicine and Community Health, University of Minnesota, Minneapolis, MN 55455, USA; 2 Department of Pharmaceutical Care and Health Systems, University of Minnesota, Minneapolis, MN 55455, USA; reid0113@umn.edu

**Keywords:** barriers, Herpes zoster, immunization, long-term care, vaccination

## Abstract

The herpes zoster vaccine is recommended for use in adults 60 years of age and older to reduce the incidence and morbidity associated with infection. Its limited uptake has been attributed to logistical barriers, but uncertain efficacy and safety in subsets of this patient population could also be contributing. The purpose of this study was to evaluate the current vaccination practices, barriers to vaccination, knowledge of vaccination reimbursement and strategies to evaluate for insurance coverage among an urban, safety net, teaching hospital, geriatric primary care provider group through a survey administered via paper and online platforms. Survey participants (*n* = 10) reported lack of availability of the vaccine in their practice settings (6/10), with half of providers (5/10) referring patients to outside pharmacies or to other practice settings (2/10) for vaccine administration. Reimbursement issues and storage requirements were perceived as major barriers by 40% (4/10) of providers, whereas 80% (8/10) of providers reported that concerns about safety and effectiveness of the vaccine were not major barriers to vaccination. Logistical barriers, rather than concerns about safety and effectiveness of the vaccine, were reported as major barriers to vaccination by a significant portion of providers. Lack of availability and reimbursement problems for practice sites allow for gaps in care. Partnership with community and long-term care pharmacies could serve as a possible solution.

## 1. Introduction

Herpes zoster (HZ) primarily burdens older adults. The incidence of HZ infections increases with age due to the loss of cell-mediated immunity to the varicella zoster virus [1]. The live attenuated HZ vaccine (Zostavax) has been shown to decrease the rate of infection and postherpetic neuralgia, its main complication. The Advisory Committee on Immunization Practices (ACIP) recommends vaccination in individuals 60 years of age and older [2]. 

While these recommendations have been in place since 2008, vaccination rates have remained low with only 24.2% of eligible individuals being vaccinated in 2013 [3]. Incidence of HZ has not decreased over time, which also suggests low vaccination rates [4]. HZ vaccination rates are much lower than influenza and pneumococcal vaccination rates and are associated with more pronounced racial and ethnic disparities [3]. Although ACIP recommendations for HZ vaccination include those living in long-term care facilities (LTCF), vaccination rates specific to this population are not commonly reported. It is likely that rates in this population are similar to overall published rates. 

Barriers to HZ vaccination may explain low vaccination rates. Hurley et al. [5] evaluated current practices and perceived barriers to HZ vaccination in a national sample of general internal and family medicine providers; however, it is unknown if providers specializing in geriatrics have the same perceptions. The purpose of this study was to assess current vaccination practices, barriers to vaccination, knowledge of vaccination reimbursement and strategies to evaluate, for insurance, coverage among a geriatric primary care provider group. There is a gap in knowledge whereby current practices and perceived barriers to HZ vaccination among geriatric primary care providers is not well understood. Since this provider population likely has the most interaction with patients for whom the HZ vaccine is recommended, understanding their practices and perceived barriers is necessary to increase vaccination rates. We found that logistical barriers, mainly reimbursement issues and storage requirements, were considered the major barriers to vaccination among survey participants, as opposed to concerns for vaccine safety and effectiveness.

## 2. Materials and Methods 

With permission, our survey was adapted from the work of Hurley et al. [5]. The original survey was shortened to include questions that were pertinent to our practice settings and to increase likelihood of participation. Our 26-item survey (supplementary file) consisted of five domains including: HZ vaccine delivery methods, perceived barriers in the delivery of the HZ vaccine, knowledge of the available reimbursement for the HZ vaccine and administration, strategies for evaluating patient insurance coverage, and current practices regarding the recommendation of the HZ vaccine. Each survey question allowed and required a single response. Survey questions regarding perceived barriers in the delivery of the vaccine and current practice used four point Likert-type responses: “Major barrier,” “Somewhat of a barrier,” “Minor barrier,” and “Not a barrier.” Knowledge of vaccine delivery methods and strategies to evaluate insurance coverage were assessed by questions with the following responses: “Agree,” “Disagree,” and “Don’t know.” Finally, questions evaluating knowledge of reimbursement were presented as multiple choice: “Medicare Part B,” “Medicare Part D,” “Not covered by Medicare,” and “Don’t know, not sure.” 

The survey was administered to advanced practice providers (APP) and geriatricians in the Extended Care Department at Hennepin County Medical Center (HCMC), an urban, teaching hospital with a recognized system of primary care clinics and community pharmacies, serving low-income, uninsured, and vulnerable populations as the county’s safety net hospital. The department consists of 17 providers (nine APP, eight geriatricians) who specialize in the care of older adults and provides care to patients in two primary care clinics, 20 long-term care facilities (LTCF), and in patients’ homes. LTCF serves as the primary practice environment for the majority of the providers within the department. HCMC providers lack access to TransactRx, a Medicare Part D billing platform for use when providing covered vaccines in clinical practices and other non-pharmacy settings, nor do the community pharmacies within the health system provide vaccinations. 

Because of the variety of practice settings in which they practice, providers were instructed to answer the questions in the context of chronic care provided in LTCF or primary care clinics. Providers were instructed to exclude their care processes for patients in transitional care units, or “short-stay units,” in LTCF as they answered questions. Providers could complete the survey in two ways. Access to an online survey (SurveyMonkey Inc.; Palo Alto, CA, USA) was provided via institution email and a paper survey was made available during a department meeting. Providers were asked to complete the survey just once, using the platform of their choice and within a three-week timeframe in March 2015. All department members received a single email reminder, regardless of which survey media was chosen. 

Online and paper survey results were pooled and evaluated using Microsoft Excel software. Descriptive statistics were used to report frequencies and averages of responses. The Institutional Review Board of the Minneapolis Medical Research Foundation approved this study and written informed consent was not required. 

## 3. Results

Overall response rate was 58% (10/17) with a slight majority completing through the online platform (6/10). The majority of providers (6/10) indicated that the vaccine is not stocked, nor administered in their practice setting. In assessing alternative methods of procurement and administration, half of providers (5/10) reported referring patients to outside pharmacies for the vaccine to be administered and very few providers (2/10) reported referring patients to another clinic or practice setting to receive the vaccine. Finally, the majority of providers (8/9) denied the use of “brown-bagging” practice, which involves having the patient purchase the vaccine at an outside pharmacy to have it administered in the provider’s practice setting. 

Reimbursement problems for practice sites and storage requirements were perceived as major barriers by 40% (4/10) of providers. Concerns about safety and effectiveness of the vaccine were not perceived as a barrier by 80% (8/10) of providers, and difficulty obtaining the vaccine was not perceived as a barrier by 70% (7/10) (Table 1). 

Fifty-five percent (5/9) of providers correctly identified Medicare Part D as providing reimbursement for the vaccine cost; however, a slight majority (5/9) reported being unsure about how vaccine administration was reimbursed. The majority of providers (7/9) reported asking patients to check with their insurance plan regarding HZ vaccine coverage. Few providers (3/9) indicated that they would ask the patient to pay for the vaccine and to independently pursue reimbursement. One-third of providers (3/9) reported having the support of office staff to assist in identifying insurance coverage by contacting the patient’s insurance. Assuming no contraindications exist, approximately 67% (6/9) of providers were very likely to recommend vaccination in patients 60 to 69 years of age while only 33% (3/9) were very likely to recommend vaccination in patients 80 years old and greater (Table 2). 

## 4. Discussion

This is among the first studies to examine HZ vaccination practices and perceived barriers among geriatric primary care providers. Similar to Hurley and colleagues [5], we found that practice site reimbursement issues represented one of the greatest perceived barriers to vaccination. Storage requirements were identified in our results as another top perceived barrier to vaccination, while in Hurley et al. this element was identified as “not a barrier at all” in the majority of participants. This may represent inherent differences in practice settings. Our data also highlights the likelihood of intrasystem differences as half of participants reported storage requirements were not a barrier for vaccine use. This speaks to the need for standardization of HZ vaccination processes within our institution. Cost concerns for patients was the greatest reported major barrier in the Hurley study, where in our study it was not as prominent, with half of providers reporting it as “somewhat a barrier.” This may be evidence of a lack of thorough understanding of the providers on HZ billing and reimbursement mechanisms and associated complexity. Since both surveys were targeted at providers and did not include patients, it is uncertain whether patients would report cost as a barrier to vaccination. 

While the question in the Hurley study allowed for more than one response, a slight majority of internal and family medicine providers reported that the vaccine is stocked and administered in their clinics. This is the opposite of our findings where the majority of providers reported that the vaccine is not stored or administered at their practice sites, despite the majority of participants indicating that obtaining the vaccine product is not the barrier. Vaccines typically stored in our primary care clinics include hepatitis A and B, influenza, pneumococcal (PCV13, PPSV23), tetanus (Td and Tdap), and typhoid. Vaccines stored in LTCFs vary. Additionally, only half of respondents indicated that they refer patients to outside pharmacies for vaccine administration. These findings suggest a gap in care whereby patients are left to determine their vaccine eligibility and seek vaccination independently. This gap in care could be addressed by encouraging and seeking collaboration between community pharmacies and clinics and long-term care pharmacies and LTCFs. Collaborations with pharmacies may be advantageous because pharmacists are knowledgeable about insurance coverage of HZ vaccine and have access to billing platforms needed to submit claims for the vaccine product and its administration. In addition, the care provider could help assist in identifying those who are eligible for the vaccination, through evaluation of past medical history and vaccination records which may not be readily available to the pharmacist or known by the patient. 

Concerns about safety and effectiveness of the vaccine were not identified as major barriers to vaccination. In addition, the majority of providers indicated that more pressing medical issues taking priority was not a barrier to vaccination. The number of providers indicating that they are ‘very likely’ to recommend the HZ vaccine decreased as the age of the patient increased, which is consistent with literature indicating the effectiveness of HZ vaccine wanes with increasing age [6]. At the same time, over half of providers still reported being somewhat likely to vaccinate patients >80 years. Evidence of provider persistence in vaccinating those >80 years may suggest that providers believe the benefit of preventative interventions for HZ is worthwhile given the clinical challenges of treating HZ and its complications. 

There are a number of limitations to the study. The small size of the study is a major limitation and results reflect institution-specific practices. Our response rate of approximately 60% is less than the Hurley et al. study which had a response rate of 72%. Our survey included providers who practice in clinics, LTCF, and in patients’ homes. While framing the evaluation in the setting of chronic care of patients, barriers may differ across these practice settings and the survey was not designed to identify these differences. As it was completed in an urban area, our study may differ from results recorded in other suburban or rural practice sites. 

## 5. Conclusions 

The results of this survey suggest that logistical barriers, such as obtaining reimbursement, are greater perceived barriers than lack of evidence supporting HZ vaccination in older adults. These results, comparable to the Hurley study [5], confirm that, regardless of specialty, providers experience barriers to vaccination that are likely influenced by their the resources and infrastructure related to their practice setting. These results also draw attention to the fact that these barriers have not changed over the seven years since Hurley and colleagues conducted their work. This study identifies opportunities for collaboration between clinics, LTCF, and pharmacies to improve HZ vaccine access to patients. Future studies may explore perceived barriers to HZ vaccine among patients to compare whether financial barriers are as prevalent among patients as they are among providers. 

An adjuvanted zoster subunit vaccine has been shown to provide an age-independent effect on reducing HZ primary disease and from the data currently available; its efficacy does not seem to wane with time [7]. This vaccine may serve as an alternative to the current HZ vaccine; however, is it uncertain if it will have the same logistical barriers as the current HZ vaccine. 

## Figures and Tables

**Table 1:** Perceived barriers in the delivery of herpes zoster vaccine.

	Responses, % (*n* = 10)			
	Major Barrier	Somewhat a Barrier	Minor Barrier	Not a Barrier
Cost concerns for patients	20	50	20	10
Reimbursement problems for my practice site	40	20	10	30
“Up-front” costs to my practice site to purchase vaccine	20	30	0	50
Storage requirements for vaccine (i.e., freezer)	40	10	0	50
Difficulty obtaining vaccine	10	20	0	70
Concerns about safety of vaccine	0	10	10	80
Concerns about effectiveness of vaccine	0	20	0	80
More pressing medical issues taking priority	10	20	50	20

**Table 2:** Provider strength of recommendation for herpes zoster vaccine ^1^.

Patient Age (Years)	Provider Responses, % (*n* = 9)			
	Very Likely	Somewhat Likely	Somewhat Unlikely	Very Unlikely
60–69	66.7	22.2	11.1	0
70–79	44.4	44.4	11.1	0
≥80	33.3	22.2	33.3	11.1

^1^ Providers were instructed to assume no contraindications exist.

## Supplementary Materials

They are available online at www.mdpi.com/2226-4787/4/4/30/s1.
