# Supplementary material for: Assessment of Perceived Barriers to Herpes Zoster Vaccination among Geriatric Primary Care Providers
Source: Pharmacy (Basel). 2016 Oct 18;4(4):30. doi: 10.3390/pharmacy4040030 (PMC5419371; doi:10.3390/pharmacy4040030)
Supplement: Supplementary file 1 [file pharmacy-04-00030-s001.pdf]

Supplementary Materials: Assessment of Perceived Barriers to Herpes Zoster Vaccination among Geriatric Primary Care Providers

Katherine Montag Schafer and Shannon Reidt

Geriatric Provider Survey–Herpes Zoster Vaccine

Goal: To assess current vaccination practices, barriers to vaccination, knowledge of vaccination reimbursement and strategies to evaluate for insurance coverage.

Instruction: Please answer the questions in the context of chronic care either in the long term care facility patients or patients seen in the clinics. These questions do not apply to the care provided to TCU patients.

Question 1:

**Herpes Zoster Vaccine Delivery Methods**<br>Please select one response for each statement.	**Agree**	**Disagree**	**Don't Know**
1. The vaccine is stocked and **administered in my practice setting**.			
2. Patients are referred to an outside pharmacy to purchase the vaccine which is then **administered in my practice setting**.			
3. Patients are referred to outside pharmacy to purchase the vaccine which is **administered at outside pharmacy**.			
4. Patients are referred to another clinic or practice setting to purchase the vaccine which is **administered at that clinic or practice setting**.			

Question 2:

**Perceived Barriers in the Delivery of Herpes Zoster Vaccine**<br>Please tell us how important the following types of barriers are to administering zoster vaccine in your practice setting.<br>Please select one response for each statement.	**Major Barrier**	**Somewhat a Barrier**	**Minor Barrier**	**Not a Barrier**
1. Cost concerns for patients				
2. Reimbursement problems for my practice site				
3. "Up-front" costs to my practice site to purchase vaccine				
4. Storage requirements for vaccine (i.e. freezer)				
5. Difficulty obtaining vaccine				
6. Concerns about safety of vaccine				
7. Concerns about effectiveness of vaccine				
8. More pressing medical issues taking priority				

Question 3:

**Knowledge of Herpes Zoster Vaccine Reimbursement**<br>Please select one response for each statement.	**Medicare Part B**	**Medicare Part D**	**Not covered by Medicare**	**Don't Know, Not Sure**
1. Vaccine cost is reimbursed by				
2. Vaccine administration is reimbursed by				

Question 4:

**Strategies to Evaluate for Insurance Coverage for Herpes Zoster Vaccine**<br>How do you evaluate for insurance coverage in eligible vaccine recipients?	**Agree**	**Disagree**
1. Ask patient to check with plan regarding coverage.		
2. Ask patient to pay for vaccine and patient to pursue reimbursement.		
3. Office staff contacts the patient's plan to identify coverage.		
4. I assume the vaccine will be reimbursed by the patient's insurance coverage.		
5. Administer the vaccine and if the patient's plan does not reimburse, the patient is billed.		

Question 5:

**Herpes Zoster Vaccine Current Practice**<br>How likely are you to recommend the herpes zoster vaccine to the following, provided no labeled contraindications exist? Please select one response for each statement.	**Very Likely**	**Somewhat Likely**	**Somewhat Unlikely**	**Very Unlikely**
1. Patients 60–69 years of age				
2. Patients 70–79 years of age				
3. Patients >80 years of age				

Question 6:

**Provider Demographics**<br>Please provide one response for each statement.	**25%**	**50%**	**75%**	**100%**
1. How much of your practice is spent providing chronic care in in a long term care facility setting?				
2. How much of your practice is spent providing chronic care in a primary care clinic setting?				
3. Please provide the number of different long term care facilities you work in:				
4. Please provide the number of different primary care clinics you work in:				

Additional Comments:

Thank you for participating.
